# Chronic Stress Effects on Tumor: Pathway and Mechanism

**DOI:** 10.3389/fonc.2021.738252

**Published:** 2021-12-20

**Authors:** Hanqing Hong, Min Ji, Dongmei Lai

**Affiliations:** ^1^ The International Peace Maternity and Child Health Hospital, School of Medicine, Shanghai Jiao Tong University, Shanghai, China; ^2^ Shanghai Key Laboratory of Embryo Original Diseases, Shanghai, China; ^3^ Shanghai Municipal Key Clinical Speciality, Shanghai, China

**Keywords:** chronic stress, neuroendocrinology, immunology, cancer, targeted drugs

## Abstract

Chronic stress is an emotional experience that occurs when people encounter something they cannot adapt to. Repeated chronic stress increases the risk of a variety of diseases, such as cardiovascular disease, depression, endocrine disease, inflammation and cancer. A growing body of research has shown that there is a link between chronic stress and tumor occurrence in both animal studies and clinical studies. Chronic stress activates the neuroendocrine system (hypothalamic-pituitary-adrenal axis) and sympathetic nervous system. Stress hormones promote the occurrence and development of tumors through various mechanisms. In addition, chronic stress also affects the immune function of the body, leading to the decline of immune monitoring ability and promote the occurrence of tumors. The mechanisms of chronic stress leading to tumor include inflammation, autophagy and epigenetics. These factors increase the proliferation and invasion capacity of tumor cells and alter the tumor microenvironment. Antagonists targeting adrenergic receptors have played a beneficial role in improving antitumor activity, as well as chemotherapy resistance and radiation resistance. Here, we review how these mechanisms contribute to tumor initiation and progression, and discuss whether these molecular mechanisms might be an ideal target to treat tumor.

## 1 Introduction

Cancer is a major public health problem and is the major cause of death worldwide. Eighteen million new cancer cases and 9 million deaths occurred worldwide in 2018 (1). The occurrence of tumors involves a multifactor and multistep process (2). Most tumors are caused by an interaction of environmental and genetic factors (3). Environmental factors mainly include biological, physical and chemical factors. Humans are exposed to these carcinogenic factors through a variety of lifestyle or occupational exposures. Moreover, in most cases, humans are not simply exposed to a single carcinogenic agent, but often to a complex mix of carcinogens through a variety of lifestyles (4). Genetic factors determine an individual’s susceptibility (5). There are at least three mechanisms by which certain individuals are susceptible to tumors. Offspring acquire mutated genes through inheritance. Genetic mutations make carriers more sensitive to environmental factors, accelerating the occurrence and accumulation of cancer events. The genetically acquired mutant genes are beneficial to the cloning and selection of tumor cells and the growth of tumor cells (6). So far, more and more evidences have shown that social stress or chronic stress promotes the occurrence and development of tumor by affecting the neuro-endocrine-immune system (7, 8).

Stress is an organism’s response to external stimuli, including physiological and psychological stimuli (9), which exert effects on the molecular, cellular, organ, and psychological levels (10). Depending on how long the stimulation lasts, stress can be divided into acute stress and chronic stress (11). Generally, acute stress is beneficial to the body, while chronic stress is harmful to the body at the psychological and physiological levels (12). Numerous clinical and *in vitro* studies have shown that chronic stress affects the initiation, progression and metastasis of various cancers through changes in the neuroendocrine system and immune system. Social stress is a potential factor for higher mortality from breast cancer in women (13). Social stress is associated with increased lung cancer morbidity and mortality. The present study confirmed that social stress can stimulate the growth of non-small cell lung cancer (NSCLC) *in vivo*, and that gamma-aminobutyric acid (GABA) inhibits this effect (14).

Chronic stress can cause changes in the neuroendocrine immune system. Disruption of neurotransmitters, stress hormones and immune cells alters the microenvironment to adapt to the occurrence and development of tumors. Recently, our research team have shown that chronic stress induces invasion and metastasis of epithelial ovarian cancer through the NE/AKT/β-catenin/SLUG axis (15) (**Table 1**). In addition, chronic stress promotes breast cancer metastasis by activating the STAT3 signal pathway through Mir-337-3p (8). Chronic stress promotes lung metastasis of circulating breast cancer cells by activating β -adrenergic signal and remodeling the premetastatic niche (16)(**Tables 1**, **2**). Chronic stress induces the release of norepinephrine, which promotes oral cancer progression through β_2_-adrenergic receptors (27). Isoprorenol promotes tumor angiogenesis by activating the PlexinA1/VEGFR2-JAK2-STAT3 signal transduction pathway within human umbilical vein endothelial cells (HUVECs), which may be a candidate target for the development of an anti-tumor angiogenesis strategy (28).

**Table 1:** Published articles on chronic stress promoting tumorigenesis and development.

Tumour type	Study	Target	Mechanism	Effect on cancer
Ovarian cancer	(15)	Macrophages	Chronic stress regulates NE/AKT/β-catenin/SLUG Axis	Tumorigenesis
Breast cancer	(16)	Mononuclear phagocyte system	Chronic psychological stress upregulates the expression of CCL2 in pulmonary stromal cells and CCR2 in monocytes/macrophages.	Metastasis
Gastric cancer	(17)	β_2_ adrenergic receptor	Stress hormones activate the ADR-β_2_ signaling pathway.	Progression and metastasis
Hepatocellular carcinoma	(18)	Splenic myeloid cells	Restraint stress augments Wnt16B/β-catenin positive feedback loop.	Progression
Skin cancer	(19)	CD4+CD8+CD25+ T cells	Chronic stress increases the numbers of CD25+ cells within tumours while decreasing the numbers of CD4+ and CD8+ cells around tumours.	Tumorigenesis

**Table 2:** Drugs targeting the neuroendocrine system and immune system.

Drug	Study	Target	Mechanism	Effect
Melatonin	15	Melatonin receptors	Anti-proliferation, anti- oxidant, antiangiogenesis, and immunoregulation effects	Inhibit metastasis of ovarian cancer
6-OHDA	16	Dopaminergic neurons	Selectively destroy dopaminergic neurons	Inhibits stress-induced lung metastasis
Propranolol	24	β adrenergic receptor	Decreased number of CD3+CD8+ T cells; reduces MDSC-based immunosuppression	Inhibits the proliferation of gastric cancer cells
ICI 118,551	52	β_2_ adrenergic receptor	Inhibits the expression of CXCR4	Inhibits the invasion of breast cancer
GABA	100	The GABA receptor	Downregulates the COX-2 protein and P-5-LOX	Inhibits the development of transplanted tumours
Phentolamine	101	α adrenergic receptor	blocking adrenergic signal	Inhibits the growth and metastasis of primary tumours
Hydrocortisone	108	Glucocorticoid-receptor	Downregulates the tumour suppressor gene BRCA1	Promotes the proliferation of breast cancer cells
Silodosin	116	α_1A_ adrenergic receptor	Decreasing the expression of ELK1, C-FOS, and NF-κB	Increased sensitivity of bladder cancer cells to chemotherapy drugs
Prazosin	117	α_1_ adrenergic receptor	Block the adrenergic signal	increased the sensitivity of prostate cancer cell lines to in vitro radiation therapy

The two main neuroendocrine systems activated by chronic stress are the hypothalamic-pituitary adrenal (HPA) axis and sympathetic nervous system (SNS). The HPA axis contains three endocrine glands (the hypothalamus, pituitary, and adrenal glands). In response to chronic stress, the hypothalamus releases corticotropin-releasing hormone (CRH), which triggers the anterior pituitary gland to secrete adrenocorticotropic hormone (ACTH) (Antoni, M. H. 2006). ACTH stimulates the adrenal gland to release stress hormones, including epinephrine (E), norepinephrine (NE), and cortisol. The SNS is one of the two main parts of the autonomic nervous system, which can promote the release of catecholamines (dopamine (DA), E, and NE) by stimulating the adrenal medulla or through neurons (12).

Chronic stress also plays an important role in immune dysfunction that affects tumor behavior (29, 30). Chronic stress selectively inhibits Th1 - and CTL-mediated cellular immunity and interferon production, which impairing immune surveillance (7). The long-term decline in immune surveillance increases the risk of cancer invasion and metastasis and reduces the effectiveness of antitumor therapy. In addition, chronic stress causes DNA damage that decreases natural killer cells and dendritic cells, thus promoting lymphatic metastasis and hematogenous metastasis. Studies have reported that sympathetic fibres innervate lymphoid organs and tissues, including the thymus, spleen, lymph nodes, and bone marrow, indicating the presence of functional interactions between neurons and the immune system (31, 32). In addition, the lymphatic system is an interconnected system of vessels, spaces, and nodes in the body which circulates lymph, which is a major source of chemokines and provides a pathway for tumor cells to escape through the body (33). Thus, the interactions of neuroendocrine system and immune system may play an important role on the occurrence and development of tumors caused by chronic stress.

An increasing number of studies indicate that adrenergic signaling plays a fundamental role in chronic stress-induced tumor growth. Adrenergic receptor inhibitors take effect through blocking adrenergic signal. Propranolol, a non-selective β antagonist, inhibits the proliferation of gastric cancer cells by reducing the number of CD3+CD8+ T cells and reducing bone marrow-derived inhibitory cells (MDSC)-based immunosuppression (20) (**Tables 2**, **3**). 6-Hydroxydopamine hydrobromide (6-OHDA) blocks adrenergic signaling through ablating sympathetic nerve function, thus inhibiting stress-induced lung metastasis (16). γ-aminobutyric acid (GABA) is a kind of inhibitory neurotransmitter, which inhibits the development of transplanted tumors by down-regulating the COX-2 protein and P-5-Lox (14). In addition, the resistance to radiotherapy and chemotherapy are often the failure of treatment and affect the patient life. It has been reported that adrenergic inhibitors combined with radiotherapy/chemotherapy can effectively reverse drug resistance and radiotherapy resistance, and improve the prognosis of cancer patients (34). This could be a potentially meaningful therapeutic strategy.

**Table 3:** Summary of adrenergic receptor antagonists.

Drugs	Molecular weight	Formula	Chemical structures	Drug category
Propranolol	259.34	C_21_H_21_NO_2_	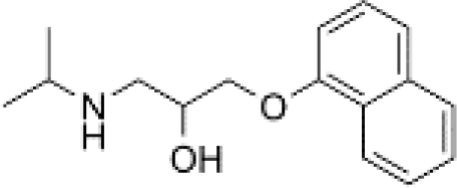	Nonselective β adrenergic receptor antagonist
Nadolol	309.40	C_17_H_27_NO_4_	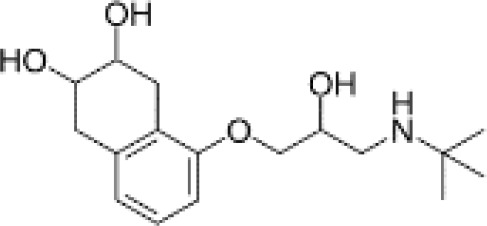	Nonselective β adrenergic receptor antagonist
(S)-Timolol Maleate	432.49	C_17_H_28_N_4_O_7_S	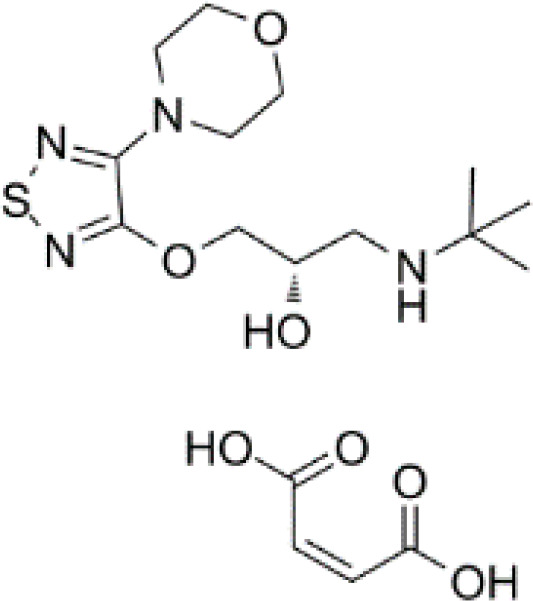	Nonselective βa drenergic receptor antagonist
Metoprolol	267.36	C_15_H_25_NO_3_	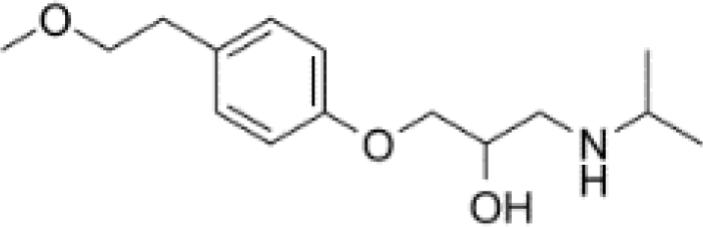	selective β_1_ adrenergic receptor antagonist
Atenolol	266.34	C₁₄H₂₂N₂O₃	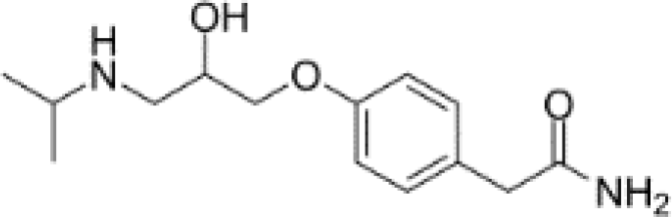	selective β_1_ adrenergic receptor antagonist
Esmolol hydrochloride	331.83	C_16_H_26_ClNO_4_	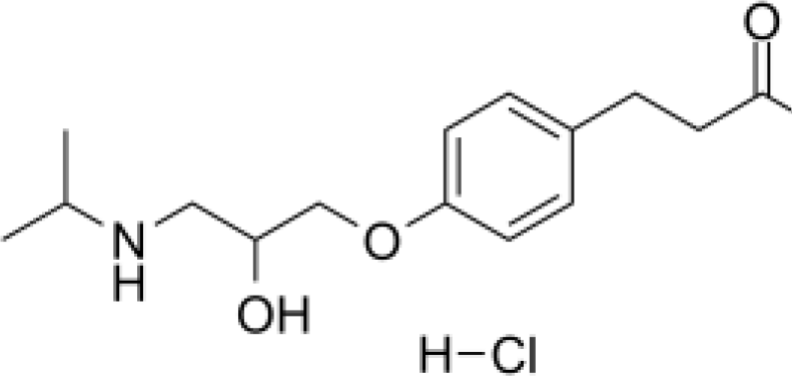	selective β_1_ adrenergic receptor antagonist
Acebutolol hydrochloride	372.89	C_18_H_29_ClN_2_O_4_	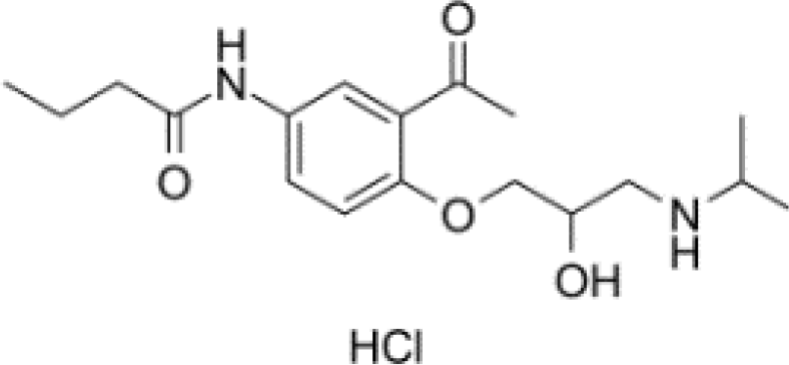	selective β_1_ adrenergic receptor antagonist
Bisoprolol	325.44	C₁₈H₃₁NO₄	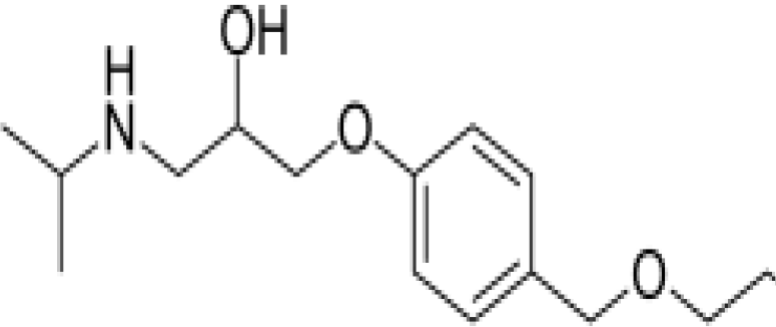	selective β_1_ adrenergic receptor antagonist
ICI 118551 hydrochloride	313.86	C_17_H_28_ClNO_2_	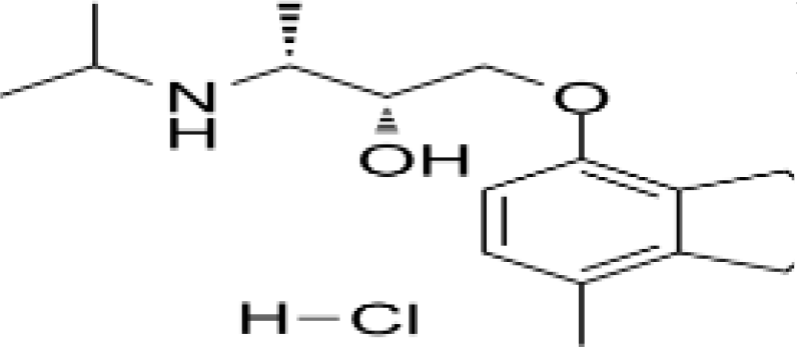	Highly selective β_2_ adrenergic receptor antagonist
Mabuterol-D9	319.80	C_13_H_9_D_9_ClF_3_N_2_O	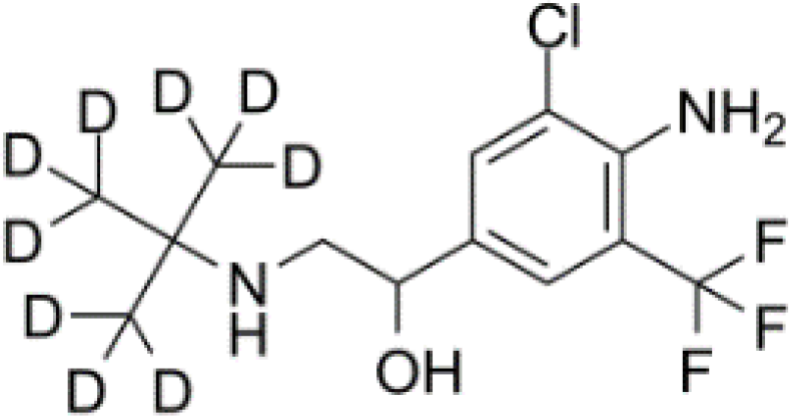	selective β_2_ adrenergic receptor antagonist
SR59230A	415.48	C_23_H_29_NO_6_	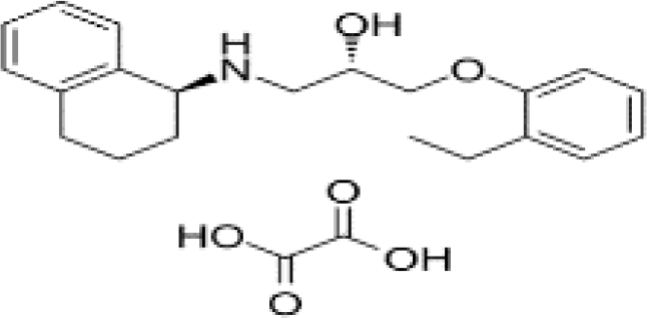	Selective β_3_ adrenergic receptor antagonists
Alfuzosin	389.45	C_19_H_27_N_5_O_4_	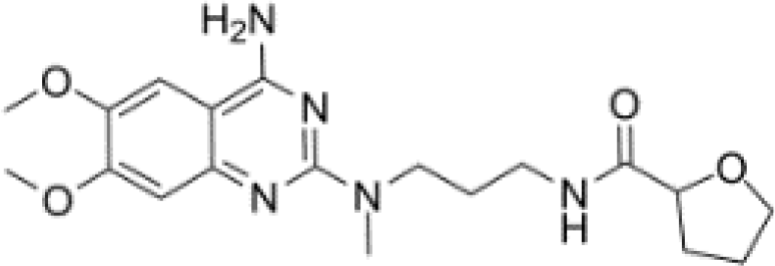	α_1_ adrenergic receptor antagonist
MG 1	303.40	C_17_H_25_N_3_O_2_	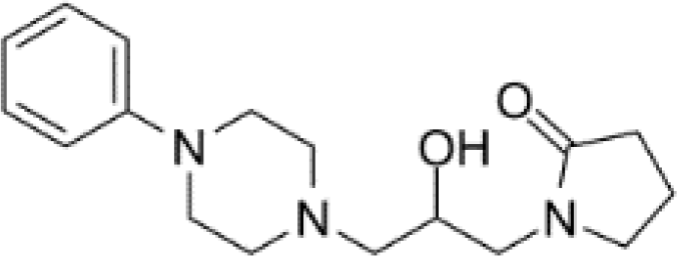	α_1_ adrenergic receptor antagonist
Yohimbine	354.44	C_21_H_26_N_2_O_3_	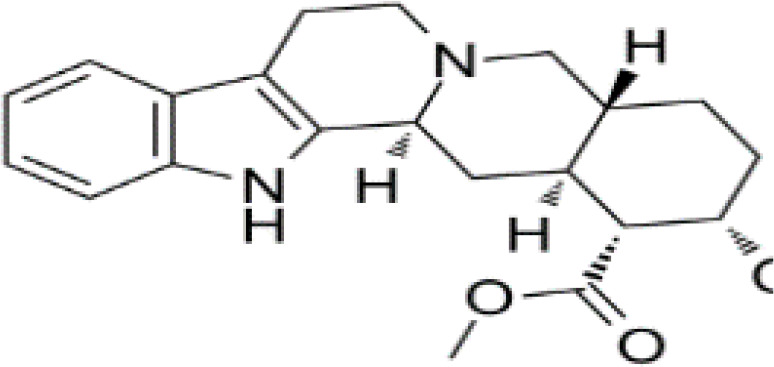	nonselective α_2_ adrenergic receptor antagonist
Rauwolscine hydrochloride	390.90	C_21_H_27_ClN_2_O_3_	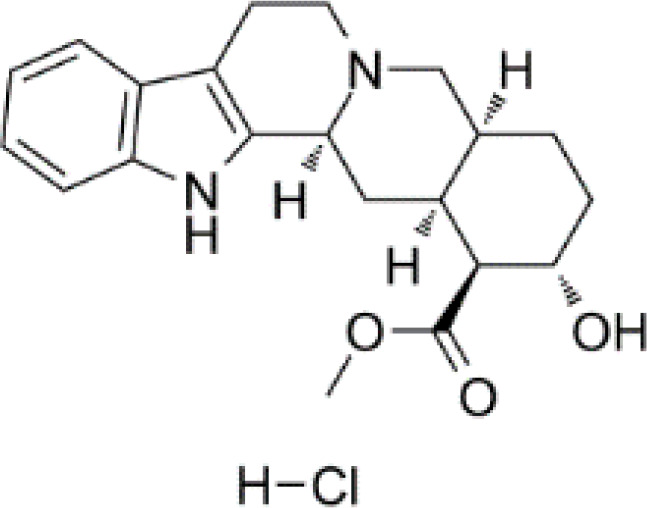	selective α_2_ adrenergic receptor antagonist
Tolazoline	160.22	C₁₀H₁₂N₂	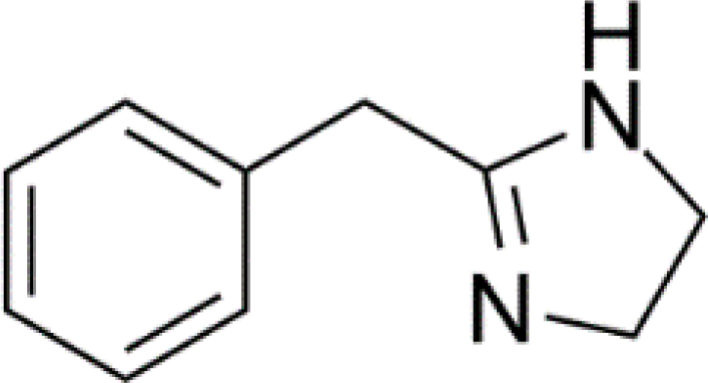	Competitive α adrenergic receptor antagonists
Phentolamine mesylate	377.46	C₁₈H₂₃N₃O₄S	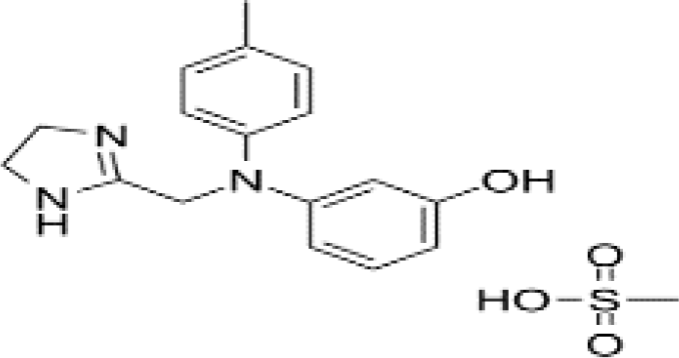	Nonselective α adrenergic receptor blockers

Here, we systematically review the role of chronic stress-mediated neuroendocrine and immune responses in the remodeling of tumor microenvironment that promotes tumorigenesis and tumor development. In addition, the inhibitory effect of adrenergic antagonists on chronic stress-related tumor development and the effect of adrenergic antagonists on chemoradiotherapy resistance will be fully discussed.

## 2 Chronic Stress Promotes Tumor Growth Through the Neuroendocrine System

### 2.1 Adrenergic Receptor Signal Pathway Induced by Chronic Stress

Chronic stress induces the release of catecholamines, which bind to adrenergic receptors (ARs) on the surface of tumor cells. Adrenergic receptors are divided into two subtypes: α and β. α-ARs are subdivided into the α_1_ and α_2_ subtypes (35). α_1_ receptors bind to the G_q_ protein and activate phospholipase C (PLC), which cleaves phospholipid phosphatidylinositol 4,5-bisphosphate (PIP_2_) into diacylglycerol (DAG) and inositol 1,4,5-trisphosphate (IP_3_). IP_3_ enters the cytoplasm and binds to IP_3_ receptors on the smooth endoplasmic reticulum (SER). The IP_3_ receptor is a calcium channel, and activation of the IP_3_ receptor leads to increased cytoplasmic calcium levels and influences a variety of intracellular events. The α_2_ receptor binds to the G_i_ protein, resulting in a decreased cAMP concentration and inhibiting protein kinase A (PKA) activity. β receptors transduce extracellular signals by binding to G_S_ proteins and activate adenylate cyclase (AC) activity, which increases the level of cAMP in the cells. Elevated cAMP results in activation of PKA. PKA induces cellular changes by altering gene expression through the phosphorylation of proteins or downstream signaling molecules or by regulating the activity of transcription factors, such as cAMP response element-binding protein (CREB) (35).

#### 2.1.1 The Activation of α-ARs

The activation of α-ARs induces cell growth by promoting cell cycle progression and preventing apoptosis (36). α-ARs may function as proto-oncogenes to promote tumorigenesis. For example, catecholamine-stimulated ARs induce tumorigenesis in the fibroblast cell line NIH3T3, suggesting the transforming potential of oncogenes and loss of contact inhibition (37). Studies have shown that adrenergic signal can promote the growth and metastasis of breast cancer by activating α-AR to enhance cell proliferation and inhibit apoptosis (38, 39). Epinephrine promotes the growth of rat pheochromocytoma PC-12 cell line by activating α_2_-AR (40). However, there have been few reports in this area.

#### 2.1.2 The Activation of β-ARs

**Figure 1:**
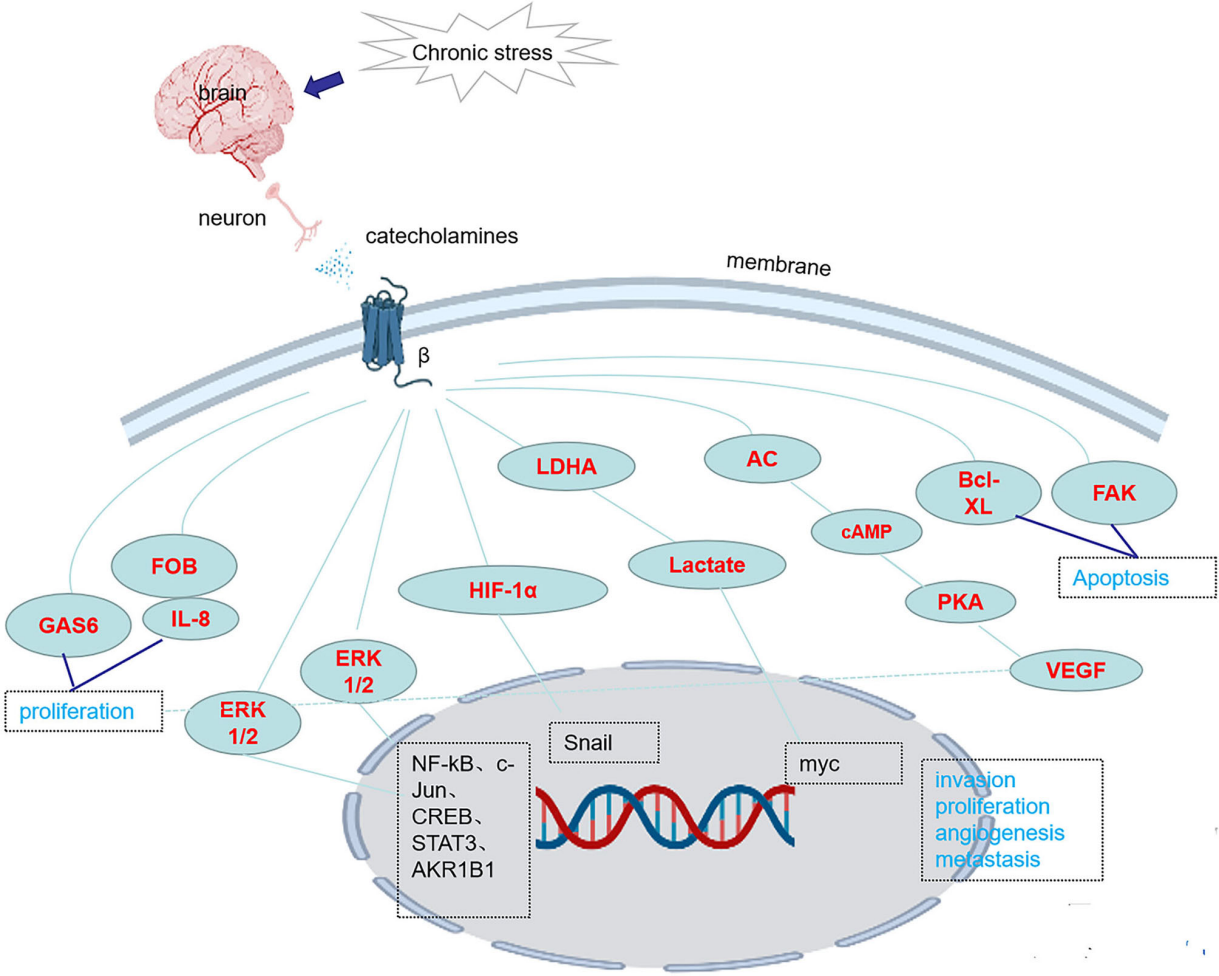
Chronic stress activates the expression of genes/proteins in related pathways through β-ARs.

There are three classes of beta receptors, β_1_, β_2_ and β_3_. Studies have shown that chronic stress causes the release of NE, which activates downstream pathways and promotes the occurrence and development of tumors by binding to β receptors, especially β_2_ and β_3_ receptor, however, the role of β_1_ receptors in tumorigenesis and tumor development has little been reported. Chronic stress induces synergistic effects on signaling through ARs, leading to the accumulation of DNA damage and promoting the development of breast cancer (41). In one study, chronic stress led to an increase in FOB-driven interleukin-8 (IL-8) through synergistic signal, which was associated with the increased growth and metastasis of ovarian cancer (42). NE induces the epithelial-mesenchymal transition (EMT) in gastric adenocarcinoma by regulating β_2_-AR-HIF-1α-Snail activity (43). NE promotes invasion and proliferation of oral squamous cell carcinoma (OSCC) by activating β_2_-AR to induce phosphorylation of extracellular regulatory protein kinase (ERK) and camp responsive element binding protein (CREB). At the same time, NE enhances the cancer stem cell -like phenotype and upregulates the expression of stem cell markers (27). Chronic stress and hormone-induced β_2_-AR activation promote breast cancer growth and VEGF/FGF2-mediated angiogenesis by downregulating PPAR (44). The β-adrenergic signal promotes tumor invasion and metastasis by altering the microenvironment of circulating tumor cells through increases in monocyte output at the premetastatic stage and macrophage infiltration into the lung (16). Catecholamine-induced β_2_-AR activation triggers shedding of Her2 by ADAM10 and subsequent intramembranous cleavage of Her2 by presenilin-dependent γ-secretase, resulting in nuclear translocation of p80 Her2 and enhanced transcription of target genes (45). Psychological stress activates the EMT through β_2_-AR, promoting tumor growth and enhancing radiation resistance (46). NE induces dormant tumor cells to enter the cell cycle by acting on osteoblasts in the tumor microenvironment (47). β_2_AR-HIF-1α-CXCL12 signaling in osteoblasts facilitates migration, invasion, and the EMT in prostate cancer cells, while β_2_-AR antagonists inhibit the effects of this pathway (48). The β_2_-AR-HIF-1α axis also regulates stress-induced pancreatic tumor growth and angiogenesis (49) (**Figure 1**). Elevated adrenaline levels activate LDHA to generate lactate *via* β_2_-AR (**Figure 1**). Changes in pH cause stabilization and ubiquitination of MYC mediated by USP28. Stabilization and ubiquitination of MYC activate the SLUG promoter, which increases the development of breast cancer (50). Isoproterenol, a β-AR agonist, regulates the release of VEGF through β-AR receptors, increasing the vascular distribution in the femurs of mice and the release of the proinflammatory cytokines interleukin-1 (IL-1) and interleukin-6 (IL-6), changing the adhesion state of endothelial cells and promoting bone metastasis of cancer cells (51). Activation of SNS pathways induced by chronic stress leads to the release of tumor-derived VEGF, which ultimately leads to lymphatic vascular remodeling and lymphatic flow, promoting tumor spread (33). Chronic stress causes the upregulation of NF-κB, CREB and STAT3, leading to gastric cancer (GC) cell proliferation and metastasis by inducing the release of NE and its binding to β-AR (17). Isoproterenol was used to simulate sympathetic nerve activation *in vivo*, and DNA strand breaks were observed in cells (52). By regulating GAS6 signaling in osteoblasts, NE induces dormant prostate cancer cells to proliferate and promotes the occurrence and development of prostate cancer (53). NE activates the PKA pathway through ARs, which induces phosphorylation of the L-type voltage-dependent calcium channel (VDCC). VDCC triggers calcium mobilization, which induces IGF-1R activation through exocytosis by insulin-like growth factor 2 (IGF2). Under chronic stress, mice with lung-specific IGF-1R expression show accelerated development of lung cancer (54). Compared with the non-stress group, the social isolation group, acute stress group, and chronic stress group showed increased CD31 expression in tumor blood vessels, which promoted tumor angiogenesis (55). NE promotes the EMT through the TGF-1/Smad3/Snail pathway and HIF-1/Snail pathway, which increase the expression of E-cadherin and vimentin and the development of tumors (48, 49). In pancreatic ductal adenocarcinoma, NE activates the Notch 1 pathway, enhances the activity and invasion of tumor cells and inhibits the apoptosis of tumor cells (56). In pancreatic cancer, β_2_-AR upregulates AKR1B1 expression, promotes proliferation and inhibits apoptosis through the ERK pathway (14)(**Table 2**). Adrenergic signaling upregulates the expression of CCL2 in lung stromal cells and CCR2 in monocytes/macrophages, leading to the recruitment and infiltration of macrophages into the lung, the formation of a premetastatic niche, and the promotion of tumor cell colonization of the lung (16) (**Table 1**). Mice transplanted with DU145 prostate cancer cells treated with NE displayed a significant concentration-dependent increase in the migration of cancer cells, which was blocked by propranolol (57). Stress neurotransmitters activate cancer stem cells (CSCs) in non-small cell lung cancer (NSCLC) through a cAMP-mediated pathway (involving VEGF, p-ERK, p-AKT, p-CREB, SHH, and ALDH-1) (58). NE induces DNA damage by interfering with the DNA repair process through the production of reactive oxygen species (ROS) and reactive nitrogen species (RNS) (59). NE reduces CXCR4 expression in MDA-MB-231 tumor cells *via* β_2_-ARs (21) (**Table 2**). Chronic stress causes the release of E and NE, activates ARs, promotes M2 macrophage polarization, increases the number of macrophages in the tumor, and regulates specific branches of the immune system (60). NE activates hematopoietic stem cells and causes them to secrete sFRP1, and sFRP1 collaborates with the Wnt16/B-catenin positive feedback loop to promote hepatocellular carcinoma (HCC) progression (18) (**Table 1**). Chronic stress causes the release of NE. After the activation of the β_2_-AR receptor by NE, the CREB-AMPK-ULk1 pathway is subsequently activated, leading to the autophagy of GC cells and resulting in the appearance of cytoplasmic vesicles in the cells. Meanwhile, the number of GFP-LC3 cells is increased, thereby promoting the proliferation and survival of GC cells (61). In addition, activation of the miR-337-3P/STAT3 axis induced by chronic stress may increase breast cancer metastasis (8).

### 2.2 The Dopamine Release Regulated by Chronic Stress

Dopamine (DA) is the neurotransmitter precursor of norepinephrine and epinephrine, and its receptor family consists of five G-protein-coupled receptors that play an important role in signal transduction (62). Dopamine has a complex effect on tumor, which can promote the occurrence and development of tumor, and inhibit the growth of tumor through the activation of different dopamine receptors. In a clinical analysis, plasma dopamine levels were significantly elevated in patients with malignant tumors. *In vitro* experiments, dopamine significantly inhibited T cell proliferation and cytotoxicity, which may be related to the intracellular cAMP elevation mediated by dopamine receptor 1(DR1). These results suggest that dopamine is involved in immune regulation (63). Chronic stress promotes blood vessel and tumor growth in a mouse model of ovarian cancer. Dopamine blocks stress-mediated tumor growth and tumor endothelial pericyte coverage by activating pericyte dopamine receptor 1 (DR1) cAMP/PKA signaling pathway (64).Dopamine receptor 2 (DR2) and hypoxia-inducible factor-1a (HIF1a) were highly expressed in tumor nuclei in stressed-induced tumor-bearing mouse models. *In vitro*, DR2 interacts with von Hippel-Lindau (VHL) in the nucleus to reduce ubiquitination mediated HIF1a degradation and enhance epithelial-mesenchymal transformation of tumor cells. Trifluoperazine (TFP), as an inhibitor of DR2, promotes the degradation of HIF1a.Thus, DR2 may promote the progression of psychological stress-induced malignancies by activating the oxygen-independent HIF1a pathway, while TFP may serve as a potential therapeutic option for cancer patients (65). In pancreatic cancer cells, inhibition of dopamine receptor 2(DR2) reduces the proliferation and migration of pancreatic cancer cells and slows the growth of xenograft tumors in mice (66). Dopamine receptor 2 agonists may be a new therapeutic option for breast cancer (67).

### 2.3 The Excess of Glucocorticoid Induced by Chronic Stress

In a mouse model of chronic unexpected mild stress (CUMS), activation of the HPA axis leads to the excessive release of glucocorticoids, which can promote the progression of liver cancer by upregulating the expression of PD-1 and inhibiting the activity of NK cells (68). The stress hormone cortisol inhibits the expression of p53 in liver cancer by increasing the expression of Bcl2 like-12 (69). Glucocorticoids induce DNA damage and interfere with the DNA repair process by inducing ROS and RNS production (59).

### 2.4 The Secretion of Oxytocin and Substance P Induced by Chronic Stress

Oxytocin (OXT) is a neurohormone produced by the hypothalamus. Oxytocin receptor (OXTRs) expression is upregulated in malignant melanoma. In addition, chronic stress can significantly increase plasma OXT levels. *In vitro*, activation of oxytocin receptor (OXTRs) promotes migration, invasion and angiogenesis of melanoma cells through the Arrestin2-dependent ERK-VEGF/MMP-2 pathway, but does not promote proliferation of melanoma cells (70). Hypothalamic oxytocin neurons regulate the progression of colitis-associated cancer (CAC) by modulating neurons in celiac-superior mesenteric ganglion (71). Oxytocin-mediated autocrine or paracrine signaling promotes the growth and development of SCCL tumors. Oxytocin antagonist as a treatment for small cell lung cancer has a certain development potential (72). Substance P, a neuropeptide, had chemotactic effect on SCCL cells (73). Substance P promotes tumor growth by promoting mitosis through its receptors (74).

## 3 Chronic Stress Promotes Tumor Growth by Affecting Immune-Related Factors

Pessimistic attitudes may be associated with decreased cytotoxicity of natural killer cells and cytotoxicity/suppressor T cells, causing squamous intraepithelial lesions and contributing to the development of cervical cancer (75). Vaccine failure in stressed mice was associated with reduced production of the effector CD8+ T cell interferons and a significant reduction in cytotoxic T lymphocyte (CTL)-mediated killing. An analysis of dendritic cell phenotypes showed that migratory and lymphoid dendritic cells were not fully mature after antigen uptake (76). Chronic stress induced a significant increase in the expression of Foxp3 and granzyme B, while social isolation significantly reduced the numbers of CD3+ and CD8+ T cells and activated CD69+ and CD3+ T cells (55) (**Table 1**). Adrenergic signaling triggered by chronic stress participates in immunosuppression of the tumor microenvironment by promoting the proliferation and activation of bone marrow-derived inhibitory cells (MDSCs) (20). Chronic stress triggers the release of stress hormones that suppress the cancer cell killing ability of granulocytes (77). Chronic stress induces the release of prostaglandins by macrophages, which in turn increases tumor cell production of VEGF, leading to vascular remodeling and lymph node metastasis (33). Chronic stress exerts a significant effect on T cell function and the production of the Th1/Th2 cell mediator H_4_R (78). Chronic stress induces Th1/Th2 imbalance in the immune system of mice and significantly promotes the progression of colon cancer (79). In chronically stressed mice, mitogen-induced T cell proliferation is reduced, the number of CD4+ T lymphocytes is reduced, and tumor necrosis factor (TNF) and interferon production are reduced, promoting tumor proliferation and progression *via* inhibition of T cell-mediated immunity (80). Thyroid hormones are important neuroendocrine regulators of tumor evolution that most likely modulate T cell-mediated immunity caused by chronic stress (80). Chronic stress may promote the progression of GC by increasing the NE-induced secretion of IL-6 in human gastric epithelial cells (81). Chronic stress reduces lymphocyte counts through TLR2-mediated PI3K signaling in a β-arrestin2-dependent manner (82). Chronic stress increases the susceptibility of a mouse model to UV light-induced squamous cell carcinoma by suppressing type 1 cytokines and protective T cells and increasing regulatory/suppressor T cell numbers (19) (**Table 1**).

## 4 Chronic Stress Promotes Tumour Growth Through the Interaction of Immunity and Neuroendocrine

Chronic stress results in dysfunctions of SNS and HPA axis. The long-term activation of SNS and HPA axes makes the immune system expose to a higher levels of stress hormones, thus disrupting the physiological internal environment (83). Activation of HPA leads to increased glucocorticoid release and activation of glucocorticoid receptor (GR). Glucocorticoids can induce DC apoptosis and inhibit DC activation and migration (84).When SNS is activated, catecholamines (epinephrine and norepinephrine) are released, which can bind to α and β adrenergic receptor receptors on immune cells (85). Catecholamines can promote macrophages to secrete pro-inflammatory factors such as IL-1β and TNF-α, intensifying the pro-tumor properties of macrophages (86). Chronic stress may stimulate the immunosuppressive activity of MDSCs and promote tumor progression (87). Activation of β_2_ receptors in TH1 cells inhibits IFN-γ production, which in turn inhibits IFN-γ -dependent B cells from producing IgG2a, thereby reducing the body’s immune capacity (88). In breast cancer, chronic stress rebuilds lymphatic networks in and around tumors through signals from the sympathetic nervous system, providing pathways for tumor cells to escape. This process is associated with macrophage COX2 inflammatory signaling and tumor-cell derived VEGFC (89). Psychological stress may induce high expression of the P53, NF-κB and p65 proteins and further promote ovarian cancer growth (90). Stress exposure decreases the TGF-β content in CD63^+^ exosomes to inhibit tumor growth. Several studies have attempted to address key mechanisms of organism reactions to stress (91). Human body is a unified organism, neuroendocrine and immunity are two very important parts of human body. Their dysfunctions provide physiological and pathological basis for the occurrence and development of tumors, and also provide ideas for the treatment of tumors.

## 5 Chronic Stress Affects the Occurrence and Development of Tumors Through Epigenetic Inheritance

Recent studies have shown that psychological and social factors can promote the development of tumors through epigenetic mechanisms (92). Epigenetic changes the expression of genes without altering the DNA sequence, including DNA methylation, histone modification, chromatin reprogramming, and non-coding RNA change (93–95). Stress hormone exposure affects the epigenetic regulation of oncogenes and tumor suppressor genes. Studies have shown that miRNA-145 is associated with chemotherapy tolerance of cervical cancer cells, and cortisol can down-regulate the expression of miRNA -145 in HPV-positive cervical cancer cells (96). Mothers with depression or anxiety had significantly increased methylation of the NR3C1 and 11β-HSD-2 genes in their placentas, which protect the fetus from maternal overexposure to stress hormones (97). Socially isolated mice had reduced expression of DNA methyltransferase (DNMT)3b and methyl CpG binding protein 2, both known epigenetic regulators (98). In a study of female ductal carcinoma in situ, high stress was associated with less histone acetylation in lymphocytes, which may influence susceptibility to tumor metastasis (99). Chronic stress induces upregulation of lysine-specific demethylase 5(KDM5A), which plays an important role in hypoxia-induced chromatin reprogramming, thereby promoting tumor progression (100). Progress has been made in the treatment of tumors, but acquired drug resistance remains an important challenge. Studies suggest that long-term exposure to stress may lead to the development of acquired resistance through epigenetic inheritance (101).

## 6 The Development of Antitumor Drugs Targeting Chronic Stress Related Tumorigenesis and Chemoradiotherapy Resistance

### 6.1 Effects of Drugs Targeting Adrenergic Receptors on Tumor Growth

Many studies report that adrenergic receptor antagonist have therapeutic effects tumorigenesis and tumor development caused by chronic stress (**Table 2**). Adrenergic receptor antagonists include α antagonist and β antagonist. α antagonists include prazosin and phentolamine. β antagonists include propranolol and metoprolol. β_2_-AR antagonists inhibit pancreatic cancer cell invasion by inhibiting CREB, NF-κB and AP-1 (102). Propranolol, a non-selective β-antagonist, reduces myeloid-derived suppressor cell (MDSC)-based immunosuppression (20).β-antagonist exhibit enhanced antiangiogenic effects under psychological stress (103) (**Table 1**).The β-antagonist propranolol inhibits adrenergic signal, a cyclooxygenase-2 (COX2) inhibitor inhibits inflammatory signaling, and a colony-stimulating factor 1 small-molecule inhibitor inhibits macrophage activity, all of which prevent chronic stress-induced lymphatic metastasis (89). Propranolol reduces the increase in Foxp3 and granzyme B levels caused by chronic stress and the decrease in the number of CD3+CD8+ T cells caused by social isolation (55). The adrenalin antagonist ICI 118,551 eliminates the effect of NE on CXCR4 expression (21). Clinically approved antihypertensive agents that block VDCC prevent the effects of chronic stress or NE on the IGF2/IGF-1R signaling cascade, as well as the transformation of lung epithelial cells and the formation of lung tumors (54). The administration of 6-OHDA to ablate sympathetic nerve function or propranolol to block adrenergic signaling significantly inhibits stress-induced lung metastasis (16). Psychological stress significantly promotes the growth of transplanted tumors, increases the levels of NE, E, cortisol, VEGF and cAMP, and decreases the levels of GABA and GAD. The reduction in cAMP levels induced by GABA therapy prevents tumor progression and signaling protein activation (104).GABA and Celecoxib downregulate the expression of the COX-2 protein and P-5-LOX, inhibits the development of xenotransplants, and reduce the systemic and tumor levels of VEGF, PGE2, and cAMP and phosphorylated signaling proteins (22). The nonselective α antagonist phentolamine inhibits the growth and metastasis of primary tumors caused by chronic stress by blocking adrenergic signal (23) (**Table 2**).

In the present study, different subtypes of adrenergic receptor antagonists also showed different effects in inhibiting tumor development. Pharmacological analysis found that SNS effects were mediated primarily by β_2_ or β_3_ adrenergic receptors in ovarian, breast, and prostate cancer models (105, 106). In these models, β_1_ receptor inhibitors, such as atenolol, generally do not inhibit the effects of SNS on tumor progression. In an epidemiological analysis of breast cancer, nonselective β antagonist have shown greater protection than β_1_ antagonist (107). In the coming years, we can expect further data expansion to evaluate the efficacy of adrenergic receptor antagonists as cancer therapy.

### 6.2 Effects of Immunomodulatory Drugs on Tumour Growth

Studies have found that chronic stress reduces antioxidant activity, leads to the accumulation of free radicals, impedes DNA damage repair and promotes the development of skin cancer (108). The involvement of free radicals in tumor initiation and development suggests that free radical scavenger may play an inhibitory role in tumor. Restraint stress facilitates the development of dimethyl benzanthracene (DMBA) induced mammary tumors by releasing β-endorphin and prolactin, However, naltrexone, an opioid receptor antagonist, exerts a beneficial effect by opposing the effect of β-endorphin on prolactin release in stressed animals (109). Melatonin (N-acetyl-5-methoxy-tryptamine), which is generally considered as pleiotropic and multitasking molecule, Secretes from pineal gland. It also has antioxidant, anti-ageing, immunomodulation and anticancer properties. Melatonin can reduce the burden of abdominal tumor by inhibiting NE/AKT/β-catenin/SLUG axis in ovarian cancer (15). It was reported that melatonin showed antioxidant potential in combating DMBA-induced skin cancer, confirming that melatonin has a preventive effect on DMBA-induced skin cancer (108). DA interferes with VEGF signals in endothelial cells, blocks angiogenesis and inhibits tumor growth (110). Hydrocortisone downregulates the expression of the tumor suppressor gene BRCA1 in breast cancer cells (24) (**Table 2**).

### 6.3 Effects of Adrenergic Receptor Antagonist on Tumour Chemoradiotherapy Resistance

Despite advances in cancer treatment, chemoradiotherapy remains the mainstay of treatment for most malignancies. Although chemoradiotherapy can prevent the development and growth of cancer, the effect of chemoradiotherapy is not as expected due to the emergence of chemoradiotherapy resistance (111). Drug resistance is the main failure factor for cancer patient and it is also an urgent problem to be solved.

Studies have found that chronic stress can cause the secretion of neurotransmitters and stress hormones. The adrenergic receptors can be divided into 2 types: α-receptors and β-receptors. They activate adrenergic receptor triggers, promote tumor growth, increase angiogenesis and promote drug resistance (112). Norepinephrine reduces anti-tumor immunity by activating AR-β of immune cells (113). Adrenergic signal increases the proportion of anti-apoptotic molecules that lead to tumor cell resistance to chemotherapy (114).

β receptor antagonists are widely used in people with cardiovascular and cerebrovascular diseases. Some studies have shown no benefit to the prognosis of cancer patients with β-antagonists, while others have suggested that they could prolong survival (112). The use of β antagonists was not associated with a reduction in lung cancer mortality (115). In an *in vitro* experimental study, nicotine promotes the growth and progression of non-small cell lung cancer, and β receptor antagonists may reduce the risk of developing non-small cell lung cancer in smokers (14). The epidermal growth factor receptor tyrosine kinase inhibitors EGFR-TKIs could delay tumor progression compared with chemotherapy (116). Studies have found that chronic stress hormones promote drug resistance to EGFR-TKIs, while the combination of β -antagonists and EGFR-TKIs may reduce drug resistance (117). In a recent retrospective cohort study, patients with advanced lung adenocarcinoma who received β-antagonists before chemotherapy had a better clinical outcome (112).

Silodosin is a selective α1 adrenergic receptor antagonist. Silodosin increased the sensitivity of bladder cancer cells to cisplatin by decreasing the expression of ELK1, C-FOS, and NF-κB. Therefore, Silodosin not only inhibits cancer cell viability and migration, but also enhances the cytotoxic activity of cisplatin against bladder cancer cell lines by inactivating ELK1 (25) (**Table 2**). Therefore, it is possible to overcome chemotherapeutic resistance in bladder cancer patients treated with cisplatin in combination with cisplatin.

Quinazoline is a kind of α -antagonist derivative. It includes prazosin, doxazosin, and terazosin. When used in combination with chemotherapy drugs used to treat prostate cancer, it has a sensitizing effect. The mechanism may be related to autophagy and apoptosis (111). *In vitro* studies, prazosin increased the sensitivity of prostate cancer cell lines to *in vitro* radiation therapy. In a retrospective study, Prostate cancer patients who took prazosin during radiation therapy had a significantly lower rate of biochemical recurrence than patients who did not. These findings indicate a 3.9-fold reduction in the relative risk of biochemical recurrence in patients who took prazosin with radiation therapy (26) (**Table 2**).

Hemangiosarcoma is a rare form of angiogenic cell carcinoma with a high mortality rate and few treatment options. Although there was an initial clinical response to chemotherapy, the results remained poor, mainly due to the development of drug resistance. *In vitro* experiments showed that the mechanism of drug resistance was that doxorubicin was a hydrophobic and weakly alkaline chemotherapy drug, which was highly accumulated in lysosomes of human hemangiosarcoma cell lines. Because its isolation in lysosomes limits its action on cellular targets, resistance develops. Propranolol is a non-selective β antagonist that contains a weakly basic amine moiety and has been shown to accumulate in lysosomes. Propranolol can reduce the accumulation of doxorubicin in in lysosomes and cell efflux, thus increasing the concentration of doxorubicin in the nucleus, making cells sensitive to doxorubicin, resulting in long-term cell stress and apoptosis (118).

Although adrenergic receptor antagonists have been reported to inhibit tumor and affect tumor resistance to chemoradiotherapy. However, there are still several problems needed to be solved (119). Firstly, the main indication for β-blockers is cardiovascular disease, and whether its side effects affect the prognosis of cancer patients needs to be evaluated. Secondly, whether it interferes with the antitumor effects of other cytotoxic drugs need to be elucidated (e.g., ACE inhibitors) (119). Therefore, current observational studies cannot guide the clinical use of β -blockers in cancer treatment, and prospective randomized controlled trials are needed to evaluate the clinical efficacy of adrenergic antagonists.

## 7 Concluding Remarks and Future Directions

Chronic stress causes systemic changes in the human body, eventually leading to changes in the neuroendocrine system and immune system. Chronic stress can activate the hypothalamic-pituitary adrenal axis and the sympathetic nervous system, cause the release of endocrine hormones and promote the occurrence and development of tumors. Activated α and β receptors can promote cell cycle progression and inhibit cell apoptosis through downstream signaling pathways. Some studies have shown that β-blockers can reduce the effects of chronic stress-induced tumorigenesis and tumor progression. Chronic stress also promotes the development of tumors by causing immune disorders in the body, which decrease the numbers of CD4+ and CD8+ cells around tumors and reduce tumor necrosis factor, interferon and macrophage levels. Attention has been given to the crosstalk between the neuroendocrine and immune systems induced by chronic stress. Chronic stress causes the release of glucocorticoids, which can promote the progression of liver cancer by upregulating PD-1 and inhibiting the activity of NK cells. β-Adrenergic signaling promotes tumor invasion and metastasis by altering the microenvironment of circulating tumor cells, inducing dormant tumor cells to enter the cell cycle, increasing the output of monocytes in the premetastatic stage and the infiltration of macrophages into the lung. In addition, adrenergic receptor blockers may improve tumor resistance to chemoradiotherapy. In order to explore its application potential, more experimental studies are necessary.

In conclusion, chronic stress can activate the hypothalamic-pituitary adrenal axis and the sympathetic nervous system, causing the release of endocrine hormones that mediate intracellular signaling pathways that promote the occurrence and development of tumors. However, the mechanism underlying the role of the neuroendocrine immune interactions induced by chronic stress in tumor pathogenesis and metastasis needs further study. In today’s society, people are under increasing chronic stress, and the adverse effect of chronic stress on tumor growth cannot be ignored. The development of antitumor drugs targeting chronic stress related tumorigenesis and chemoradiotherapy resistance might be a new strategy of cancer therapy.

## Author Contributions

DML, HQH was involved in data acquisition, analysis and manuscript drafting. DML and MJ revised the manuscript. All authors contributed to the article and approved the submitted version.

## Funding

This study was funded by the National Key Research and Developmental Program of China (2018YFC1004800 and 2018YFC1004802), the Shanghai Municipal Council for Science and Technology (18410721200 and 20JC1412100), and the National Natural Science Foundation of China (81971334).

## Conflict of Interest

The authors declare that the research was conducted in the absence of any commercial or financial relationships that could be construed as a potential conflict of interest.

## Publisher’s Note

All claims expressed in this article are solely those of the authors and do not necessarily represent those of their affiliated organizations, or those of the publisher, the editors and the reviewers. Any product that may be evaluated in this article, or claim that may be made by its manufacturer, is not guaranteed or endorsed by the publisher.

## Abbreviations

HPA, hypothalamic-pituitary adrenal; SNS, sympathetic nervous system; CRH, corticotropin-releasing hormone; ACTH, adrenocorticotropic hormone; E, epinephrine; NE, norepinephrine; DA, dopamine; ARs, adrenergic receptors; AC, adenylate cyclase; TNF, tumor necrosis factor; cAMP, Cyclic adenosine monophosphate.
